# Finger-Prick Whole Blood Cryptococcal Antigen Lateral Flow Assay for the Diagnosis of Cryptococcosis in HIV-Negative Patients: A Case Series Study in Two Tertiary Centers in São Paulo, Brazil

**DOI:** 10.3390/jof9121140

**Published:** 2023-11-25

**Authors:** José E. Vidal, Fernanda Gurgel Oliveira, Marcela Vieira, Luisa Pereira, Rodovaldo M. Lucas Junior, Bruno Fukelman Guedes, Marcello Chaves Magri, David R. Boulware

**Affiliations:** 1 Departamento de Neurologia, Instituto de Infectologia Emílio Ribas, São Paulo 01246-900, Brazil; 2 Departamento de Moléstias Infecciosas e Parasitárias, Hospital das Clínicas da Faculdade de Medicina da Universidade de São Paulo, São Paulo 05403-000, Brazil; marcello.magri@hc.fm.usp.br; 3 Laboratório de Investigação Médica (LIM 49) da Faculdade de Medicina da Universidade de São Paulo, São Paulo 05403-000, Brazil; 4 Departamento de Infectologia, Instituto de Infectologia Emílio Ribas, São Paulo 01246-900, Brazil; fernandagurgelo@gmail.com (F.G.O.); marcelavieiraf@gmail.com (M.V.); luisaaa.pereira@gmail.com (L.P.); lucasrodovaldo13@gmail.com (R.M.L.J.); 5 Departamento de Neurologia, Hospital das Clínicas da Faculdade de Medicina da Universidade de São Paulo, São Paulo 05403-000, Brazil; bruno.guedes@hc.fm.usp.br; 6 Division of Infectious Diseases and International Medicine, Department of Medicine, University of Minnesota, Minneapolis, MN 55455, USA; boulw001@umn.edu

**Keywords:** cryptococcosis, cryptococcal meningitis, diagnosis, point-of-care testing, lateral flow assay, *Cryptococcus gattii*, Brazil

## Abstract

Cryptococcosis in HIV-negative patients can be an opportunistic or endemic disease. There are no published studies on the use of the finger-prick whole blood (point-of-care) cryptococcal antigen lateral flow assay (CrAg LFA) for diagnosing cryptococcosis in HIV-negative patients. We conducted a case series study of HIV-negative patients with cryptococcosis in two centers in São Paulo, Brazil. The objectives were to identify the sensitivity of a finger-prick whole blood CrAg LFA and to describe the main characteristics of this population. We identified 30 HIV-negative patients with cryptococcosis [19 (63%), male; median age, 47 years]. Ten (33%) patients were immunosuppressed, ten (33%) had other comorbidities, and ten (33%) were apparently immunocompetent and without comorbidities. The distribution of the sites of cryptococcosis was as follows: the central nervous system, 90% (n = 27); pulmonary, 43% (n = 13); and other extrapulmonary sites, 40% (n = 12). The sensitivity of the finger-prick whole blood CrAg LFA for the diagnosis of cryptococcosis was 97% (29/30). Among 26 participants with cryptococcal meningitis, the sensitivity of testing cerebrospinal fluid was as follows: CrAg latex agglutination, 77% (20/26); CrAg LFA, 96% (25/26); and culture, 81% (21/26). Culture speciation identified *Cryptococcus gattii* in 16 (62%) cases, and all had a positive finger-prick whole blood CrAg LFA. This test presented high sensitivity to the diagnosis of cryptococcosis in HIV-negative patients, including those caused by *C. gattii.*

## 1. Introduction

Cryptococcosis is caused by encapsulated yeast of the *Cryptococcus neoformans*/*Cryptococcus gattii* species complexes [1]. *C. neoformans* has a worldwide distribution and has been classically identified as an opportunistic fungus that frequently causes meningitis in people living with HIV/AIDS (PLWHA) [2]. However, the frequency of cases of cryptococcosis in HIV-negative patients is increasing due to the presence of several subsets of patients with impaired cell-mediated immunity, including the use of immunosuppressive therapies and monoclonal antibodies, solid organ transplantation, malignant and lymphoproliferative disorders, rheumatologic diseases, end-liver disease, and end-kidney disease [3]. In contrast to *C. neoformans*, *C. gattii* has a more limited geographic distribution, particularly in mainland Australia, and tropical/subtropical areas in Central Africa, northern Australia and Central and South America, but outbreaks of the disease have been described in the temperate areas of British Columbia, Canada and expanded to the Pacific Northwest region of Canada and the United States [4], and typically cause disease in apparently immunocompetent hosts [5,6,7,8]. 

Cryptococcosis was the main cause of mortality among all AIDS-associated systemic mycoses and the second most common cause of mortality among systemic mycoses in the general population in a review from 1996 to 2006 in Brazil [9]. Furthermore, approximately 25% of patients who died from cryptococcosis between 2000 and 2012 in Brazil did not have HIV infection, with 5% having other risk factors for cryptococcosis and 15% having no report of any diagnosis predisposing to cryptococcosis [10]. 

The distribution of the molecular types of *C. neoformans*/*C. gattii* species complexes showed a regional pattern in Brazil, where *C. gattii* predominantly occurs in the Northern region, and *C. neoformans* predominantly occurs in the Southern macro region [10,11]. In this complex epidemiological scenario, the mortality of cryptococcosis continues to be unacceptably high in Brazil [12], and timely and accurate diagnosis is a key strategy to improve the outcome of the different groups of patients who have cryptococcosis. 

Recently, the World Health Organization (WHO) published the Fungal Priority Pathogens List (FPPL) [13]. This document classified fungal pathogens into three categories: critical, high, and medium priority. Interestingly, *C. neoformans* was included as one of the four fungal pathogens of the critical priority group, and *C. gattii* was included as one of the eight fungal pathogens of the medium priority group [13]. 

Over the last decade, the availability of a cryptococcal antigen lateral flow assay (CrAg LFA) (Immuno-Mycologics Inc., Norman, OK, USA, IMMY), an immunochromatographic rapid test, has been a game changer in the diagnosis of cryptococcal meningitis in PLWHA. The WHO recommends the use of cerebrospinal fluid (CSF) CrAg LFA as the preferred diagnostic assay for HIV-related cryptococcal meningitis [14]. In these patients, the CrAg LFA can be performed in settings with minimal or no infrastructure. The IMMY CrAg LFA can be additionally used in serum, plasma, and whole blood specimens [15]. In contrast to serum, whole blood constitutes a real point-of-care sample due to its bedside availability. The finger-prick whole blood CrAg LFA has demonstrated good performance for the diagnosis of cryptococcal meningitis in PLWHA [16,17], but in the case of screening asymptomatic patients, the use of a transfer pipette seems to optimize its yield [18].

Some studies have evaluated the performance of serum CrAg LFA in HIV-negative patients with cryptococcosis [19,20], but information on the performance of finger-prick whole blood CrAg in this population is still limited but necessary due to the potential benefits of its implementation in urban and rural settings in low- and middle-income countries. 

We conducted a study to evaluate the use of finger-prick whole blood CrAg LFA among HIV-negative patients with cryptococcosis in São Paulo, Brazil. 

## 2. Patients and Methods

### 2.1. Study Design

This was a case series study where we evaluated the sensitivity of IMMY (Immuno-Mycologics Inc., Norman, OK, USA) CrAg LFA on finger-prick whole blood collected from HIV-negative patients with proven cryptococcosis.

### 2.2. Setting Study

This study was conducted at the *Instituto de Infectologia Emílio Ribas* (IIER) and *Hospital das Clínicas*, *Faculdade de Medicina*, *Universidade de São Paulo* (HC-FMUSP), both tertiary centers in São Paulo State, Brazil, between January 2017 and December 2022.

### 2.3. Study Participants

Patients with the following criteria during hospital admission were included: (i) non-reactive HIV serology; (ii) proven cryptococcosis; and (iii) the result of finger-prick whole blood CrAg LFA. 

Non-reactive HIV serology followed the criteria from the Ministry of Health of Brazil. Proven cryptococcosis followed the criteria from the European Organization for Research and Treatment of Cancer and the Mycoses Study Group Education and Research Consortium: (i) histopathologic, cytopathologic, or direct microscopic examination of a specimen obtained via needle aspiration or biopsy from a normally sterile site (other than mucous membranes) showing encapsulated budding yeasts compatible with the *Cryptococcus* species; (ii) recovery of yeast by culture from a sample obtained by a sterile procedure from a normally sterile site showing a clinical or radiological abnormality consistent with cryptococcosis; (iii) a blood culture that yields the *Cryptococcus* species; (iv) a cryptococcal antigen in the CSF or blood; and (v) the amplification of *Cryptococcus* species DNA via a PCR combined with DNA sequencing when yeasts are seen in formalin-fixed paraffin-embedded tissue [21]. 

Sites of cryptococcosis were classified in the pulmonary, central nervous system, and other extrapulmonary sites [21]. Pulmonary cryptococcosis was defined as patients with a cryptococcal disease involving the lungs with or without another category. Central nervous system cryptococcosis was defined as the presence of cryptococcal meningitis and/or cerebral cryptococcoma with or without another category. 

Other extrapulmonary sites included those without pulmonary or central nervous system involvement (i.e., skin, disseminated blood). All patients included in this study had a unique diagnosis that fell into one of the categories cited. The diagnostic criteria of disseminated cryptococcosis are defined as two or more non-adjacent organs that are simultaneously affected by cryptococcosis.

Patients were categorized into the following three groups based on their underlying diagnosis: (i) immunosuppressed patients; (ii) patients with other comorbidities; and (iii) apparently immunocompetent patients and those without comorbidities. Immunosuppressed patients were defined as those with malignant and lymphoproliferative disorders, solid organ transplantation, sarcoidosis, rheumatologic diseases, end-stage liver disease, end-stage kidney disease, primary immunodeficiency, or receiving corticosteroids, immunosuppressive therapies, and/or monoclonal antibodies [2]. Patients with other comorbidities were defined as those with any other disease or condition not included in the definition of immunosuppressed patients (e.g., chronic cardiovascular diseases, chronic respiratory diseases, diabetes mellitus, obesity, tuberculosis). Diabetes mellitus has not been clearly defined as a risk factor for cryptococcosis [2]. Apparently, immunocompetent patients and those without comorbidities were defined as not classified in the previous two groups.

### 2.4. Study Procedures

The medical charts of the individuals included in this study and laboratory electronic databases were reviewed. The CrAg LFA on finger-prick whole blood was conducted at the point of care in the recruiting clinics. JEV, FG, MV, RMLJ, and LP performed the tests as part of the clinical routine. The finger-prick whole blood sample was collected by pricking the pad of the index finger with a lancet, following the disinfection of the finger with an alcohol swab. The droplet of blood was applied directly onto the sample pad of a CrAg LFA test device. The device was immediately immersed into a drop of sample diluent in a tube and removed 10 min later for interpretation, as guided by the manufacturer. If a lumbar puncture was performed by the attending physician, IMMY CSF CrAg LFA testing was offered because this test was not available in routine care where this study was carried out. The results of finger-prick whole blood and CSF CrAg LFA were reported to the attending physicians for further investigation and treatment at their discretion. The patients received the standard management for cryptococcosis at each institution where individuals admitted for suspected cryptococcosis routinely underwent lumbar puncture, increased intracranial pressure was aggressively addressed, and combination antifungal therapy was the standard of care. 

### 2.5. Statistical Analysis

Descriptive statistics were used. Frequency, median, and interquartile rates (IQR) were calculated using Microsoft Excel 2013.

### 2.6. Ethical Statements

The Research Ethics Committee of IIER and HC-FMUSP approved this study. The patients’ data were anonymized/de-identified to protect patients’ privacy/confidentiality. 

## 3. Results

### 3.1. Demographic, Clinical, Neuroradiological and Outcome Characteristics 

Thirty patients were included in this study. Nineteen (63%) cases were males. The median age of participants was 47 (IQR, 39–57) years. No participant had a history of prior cryptococcosis. Among the 25 patients with available information, the median duration from symptom onset to the diagnosis of cryptococcosis was 30 (IQR, 21–75) days. Ten (33.3%) patients were classified as immunosuppressed as follows: solid organ transplantation (kidney, n = 2; kidney-pancreas, n = 1 cardiac, n = 1), liver end-disease (n = 2), kidney end-disease (n = 1), and primary immunodeficiency (n = 3). Twenty (66.6%) patients did not have any disease or condition classified as immunosuppressive; 10 (33%) patients had other comorbidities, including hypertension (n = 5), diabetes mellitus type 2 (n = 3), alcoholism (n = 2), obesity (n = 1), atrial tachycardia (n = 1), gout (n = 1), cocaine addiction (n = 1), tuberculosis (n = 2), traumatic spinal cord injury (n = 1); and 10 (33%) patients were classified as apparently immunocompetent and without comorbidities. Table 1 shows the main features of the non-HIV individuals with cryptococcosis included in this study. At admission, the patients presented the following clinical manifestations: neurological, n = 27 (90%); pulmonary, n = 3 (10%); and other extrapulmonary sites, n = 3 (skin, n = 2; and lymph nodes, n = 1). Twenty-eight (93%) patients had brain magnetic resonance imaging performed, and only four (13%) had normal findings. The other 24 (87%) participants had presumed cryptococcosis-related lesions as follows: leptomeningeal enhancement, n = 15 (62.5%); hydrocephalus = 9 (37.5%); dilated Virchow–Robin spaces, n = 7 (29%); mucinous pseudocysts, n = 7 (29%); ependymal enhancement, n = 6 (25%); cryptococcoma, n = 5 (21%); and vasculitis, n = 1 (4%). All participants received combination antifungal therapy as an induction: amphotericin B deoxycholate plus flucytosine, n = 9 (30%); lipid complex amphotericin plus flucytosine, n = 8 (27%); amphotericin B deoxycholate plus fluconazole, n = 4 (13%); liposomal amphotericin B plus flucytosine, n = 4 (13%); lipid complex amphotericin plus fluconazole = 3 (10%); and liposomal amphotericin B plus fluconazole = 2 (7%). Six (20%) participants died during hospitalization, and 24 (80%) were discharged to home.

### 3.2. Laboratory Results and Diagnosis of Cryptococcosis

The distribution of the sites of proven cryptococcosis was as follows: central nervous system, n = 27, 90%; pulmonary, n = 13, 43%; and other extrapulmonary sites, n = 12, 40% (skin, n = 2; lymph nodes, n = 2; genitourinary system, n = 2; blood, n = 2; kidney, n = 1; adrenal, n = 1; bone, n = 1, ocular, n = 1). All cases of pulmonary cryptococcosis had a concomitant central nervous system and/or disseminated cryptococcosis (no patient had isolated pulmonary cryptococcosis).

The sensitivity of the finger-prick whole blood CrAg LFA in the diagnosis of cryptococcosis was 97% (29/30). The single case with negative finger-prick whole blood CrAg LFA was a patient with end-stage kidney disease and cerebral cryptococcomas (Case 7). The sensitivity of serum CrAg latex agglutination in the diagnosis of cryptococcosis was 57% (12/21). 

**Figure 1:**
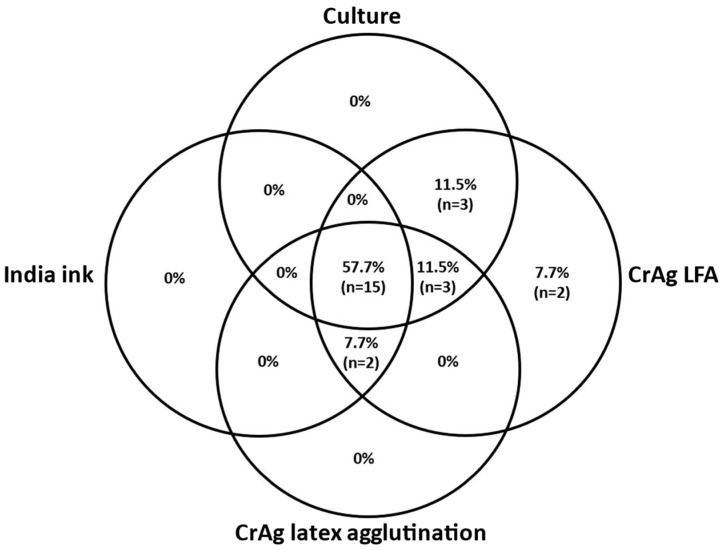
Venn diagram of distribution of 26 non-HIV individuals with cryptococcal meningitis tested with India ink, culture, cryptococcal antigen latex agglutination, and a cryptococcal antigen lateral flow assay in cerebrospinal fluid samples. Notes. CrAg: cryptococcal antigen; LFA: lateral flow assay.

Among 27 patients with proven central nervous system cryptococcosis, 26 (96%) cases had meningitis, and one (4%) had a single cryptococcoma without meningitis. Three (10%) participants had no diagnosis of central nervous system cryptococcosis: meningitis was ruled out in one case, and the other two participants had contraindications to perform a lumbar puncture. Among the 26 participants with proven cryptococcal meningitis, the sensitivity of the CSF tests was as follows: India ink, 65.4% (17/26); CrAg latex agglutination, 76.9% (20/26); CrAg LFA, 96.2% (25/26); and culture, 80.8% (21/26). Fifteen (57.7%) participants had positive or reactive results in all these four tests. CSF CrAg LFA was the only positive test in two (7.7%) cases, which also tested positive in finger-prick whole blood CrAg LFA. Figure 1 shows the distribution of non-HIV individuals with cryptococcal meningitis tested with India ink, culture, cryptococcal antigen latex agglutination, and CrAg LFA in CSF samples. 

**Figure 2:**
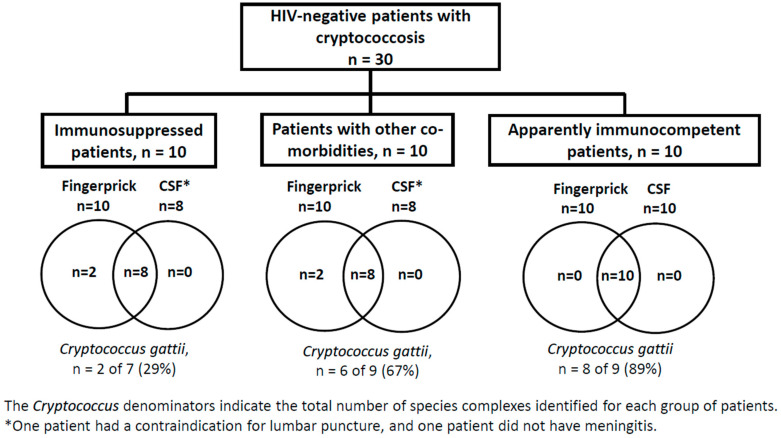
Distribution of study participants according to host type; results of the cryptococcal antigen lateral flow assay in whole blood and cerebrospinal fluid; and *Cryptococcus* species complex. Notes. Lateral flow assay was performed in whole blood of 30 patients and in cerebrospinal fluid of 28 patients; CSF: cerebrospinal fluid.

The *cryptococcus* species complex was identified in 86.7% (26/30) participants: *C. neoformans* in 38.5% (10/26) and *C. gattii* in 61.5% (16/26). In these patients, the sensitivity of finger-prick whole blood CrAg LFA for the diagnosis of cryptococcosis was 100%. Among the four (13.3%) participants with negative fungal cultures, three cases (Cases 3, 9, and 18) had positive finger-prick whole blood CrAg LFA, and one had negative finger-prick whole blood CrAg LFA (Case 2). Figure 2 shows the distribution of the study participants according to the host type, results of the CrAg LFA, and *Cryptococcus* species complex. 

The three cases with cryptococcosis but without neurological manifestations at admission had an isolation of *C. gattii*: one had subclinically proven cryptococcal meningitis (Case 22), one had no cryptococcal meningitis (Case 11), and one did not have a lumbar puncture performed (Case 12).

## 4. Discussion

In this study, the finger-prick whole blood CrAg LFA presented high sensitivities in HIV-negative patients with proven cryptococcosis (97%), cryptococcal meningitis (96%), and cryptococcal meningitis caused by *C. gattii* (100%). To the best of our knowledge, this is the first study to evaluate the sensitivity of finger-prick whole blood CrAg LFA in HIV-negative patients with proven cryptococcosis.

The clinical presentation of cryptococcosis had a broad spectrum of systemic manifestations, particularly neurologic and pulmonary [2,3,5,22], progressing over weeks with potential overlap with other diseases affecting HIV-negative patients. In the present study, 90% of patients had proven central nervous system cryptococcosis, and this finding highlights the importance of the syndromic approach to cryptococcosis. In Brazil and in other similar scenarios, the presence of subacute or chronic meningitis/meningoencephalitis should prioritize the investigation of cryptococcosis as the main differential diagnosis of tuberculosis [22].

The diagnosis of cryptococcosis in HIV-negative patients is challenging, particularly in resource-limited settings where laboratory-based diagnosis continues to be restricted [23]. The availability of a laboratory diagnosis in Latin America and the Caribbean is usually scarce, allocated inefficiently, and concentrated in urban areas [24]. However, several tertiary referral centers have experience with conventional mycologic methods, including the culture of clinical specimens or demonstration of yeast on cytopathology or histopathology examination.

The World Health Organization characterizes an ideal point-of-care diagnostic as a device with the ASSURED criteria. The finger-prick whole blood CrAg LFA fits these criteria for the diagnosis of cryptococcal meningitis and is a true POC test: Affordable, Sensitive, Specific, User friendly, Rapid/robust, Equipment-free, and Deliverable. International IMMY document indicates that CrAg LFA can use whole blood (venous and finger-prick) [15], but this sample type is not yet Food and Drug Administration-approved inside the United States. A study reported 100% agreement between whole blood, serum, and plasma IMMY CrAg LFA in PLWHA with cryptococcal meningitis, suggesting that finger-prick whole blood CrAg is a reliable bedside diagnostic test [16]. A study in HIV-related CrAg screening identified that finger-prick whole blood IMMY CrAg LFA resulted in reduced sensitivity (~80%) when compared to the reference standard of laboratory-based CrAg LFA on plasma among a population with low CrAg titers. This study identified that the use of a pipette to transfer and mix finger-prick whole blood with the diluent prior to testing and reading the result after 20 min increased sensitivity to 100% [18]. Despite the possibility of pipette use implementation in resource-limited settings [25], this step is not necessarily user-friendly. A recent study evaluated the performance of finger-prick whole blood IMMY CrAg LFA clinic-based testing compared to serum IMMY CrAg laboratory-based testing in PLWHA. This test showed sensitivity, specificity, a positive predictive value, and a negative predictive value of 91.7%, 99.5%, 64.7%, and 99.9%, respectively, in PLWHA with cryptococcal meningitis [17]. In the same study, when symptomatic and asymptomatic cryptococcal disease were concomitantly analyzed, the finger-prick whole blood IMMY CrAg LFA showed sensitivity, specificity, a positive predictive value, and negative predictive value of 48%, 99.5%, 91%, and 94.7%, respectively, and the authors concluded that whole blood should not be used to screen for asymptomatic cryptococcal disease in advanced HIV disease [17]. In the case of HIV-negative patients, the strategy to screen for cryptococcal disease is not a concern. Taken together, the available information about the use of finger-prick whole blood CrAg LFA in PLWHA suggests its potential use in HIV-negative patients.

The CrAg LFA test has been evaluated in serum and CSF samples but not in whole blood from HIV-negative patients. Recently, a systematic review to characterize the diagnostic performance of IMMY CrAg LFA in HIV-negative populations on serum and CSF was published [26]. Eight studies evaluated the diagnostic performance of the CrAg LFA on serum, and the pooled median sensitivity was 96% [95% Credible Interval (CrI) 68–100%] with a pooled specificity estimate of 96% (95% CrI 84–100%). In addition, six studies evaluated the diagnostic performance of CrAg LFA on CSF, and the pooled median sensitivity was 99% (95% CrI 95–100%) with a pooled specificity median of 99% (95% CrI 95–100%). In this review, the high sensitivity and specificity of CrAg LFA in serum and the CSF of HIV-uninfected people was similar to previously reported values in studies on PLWHA. However, the small number of studies, their unclear or high risk of bias, their significant heterogeneity, and/or the presence of limited data prevented any further sub-analyses regarding the performance of CrAg LFA among different groups of patients or between different cryptococcal species. Despite these limitations, available information suggests that serum CrAg LFA is highly useful in HIV-negative patients, particularly those with central nervous system, disseminated disease, or diffuse pulmonary cryptococcosis [19,27,28]. A recent study, not included in the systematic review cited above [26], evaluated 426 HIV-negative patients with cryptococcosis in 46 Australian and New Zealand hospitals [29]. Two hundred and fifty-nine (60.8%) patients had at least one known underlying immunocompromising condition or disease, and 167 (39.2%) patients had none of them. Serum CrAg testing (IMMY CrAg LFA in 89% of hospitals) was positive in 85.1% of patients (319 of 375 tested) [29]. By contrast, we identified a 96.7% positivity using whole blood, a finding that can be explained, at least in part, due to the more frequent central nervous system involvement in our study.

Pulmonary cryptococcosis is uncommonly diagnosed in PLWHA but is identified among 38–78% of autopsies of PLWHA with disseminated cryptococcosis [30]. In addition, isolated cryptococcal pneumonia is less frequent even in autopsy studies (e.g., 7% in autopsies of HIV-infected patients with cryptococcosis) [30]. By contrast, isolated pulmonary cryptococcosis has been more commonly described in some populations of immunocompromised hosts (e.g., solid-organ transplantation), particularly in immunocompetent individuals [30,31]. However, we did not have any cases of isolated pulmonary cryptococcosis in the present study. This finding is not fully understood, but there is likely a delay in the diagnosis of cryptococcosis, as suggested by the long median duration (30 days) from symptom onset to the confirmation of diagnosis of this fungal disease. The present study was carried out in two tertiary urban centers, and it is common that HIV-negative patients with cryptococcosis, particularly those apparently immunocompetent, seek primary care services in the first days or weeks after clinical manifestations begin, and the hypothesis of cryptococcosis has not been considered. A current challenge is to avoid missing opportunities in areas endemic for cryptococcosis, remembering to include this hypothesis in the differential diagnosis of patients with respiratory and/or neurological manifestations and considering the performance of finger-prick whole blood CrAg LFA as a true point-of-care test. However, this result should always be interpreted with caution since the sensitivity of serum CrAg has been variable in cases of isolated cryptococcal pneumonia. For instance, serum CrAg may be negative in an immunocompetent patient with mild disease (e.g., single, small pulmonary cryptococcoma), but the probability of a positive test increases in another immunocompetent patient with severe disease (i.e., bilateral cryptococcal pneumonia) [32]. Negative results need to be carefully evaluated, as reported in a recent study of HIV-negative patients with cryptococcosis, where 75% (42 of 56) of cases with a negative serum CrAg had isolated pulmonary cryptococcosis [29].

In the present study, most cases with the identification of the *Cryptococcus* species complex had central nervous system cryptococcosis caused by *C. gattii*. This fact may explain the high sensitivity of finger-prick whole blood CrAg LFA when compared to serum latex agglutination, which was designed to identify the polysaccharide capsule antigen of *C. neoformans.* IMMY CrAg LFA uses two monoclonal antibodies impregnated onto an immunochromatographic test strip to detect CrAg for *C. neoformans*/*C. gattii* species complexes [33]. This particular composition may justify the better analytical sensitivity for these two species complexes [15]. The experience using the LFA to diagnose *C. gattii* disease is limited [26], but it seems to perform similarly or better when compared with *C. neoformans* disease [5,29]. For example, a study reported that serum CrAg was positive in 77.1% of patients with *C. neoformans* disease compared with 97.3% of those with *C. gattii* (*p* < 0.001) [29].

In the present study, all cases of cryptococcosis in HIV-negative patients were the first episodes of this fungal disease, including the subset of known immunosuppression. Subsequent episodes of cryptococcal meningitis are a potential situation in immunosuppressed hosts, particularly in advanced HIV disease. In these cases, positive CrAg LFA may remain positive for months or years, and this test is not useful for the diagnosis of subsequent episodes of culture-positive relapse cryptococcal meningitis, where a positive CSF culture for *Cryptococcus* is the typical test to confirm mycological relapse. The polymerase chain reaction can also be useful to exclude relapse [34].

This study had some limitations. First, the observational and retrospective methodology defines intrinsic limitations to the design. However, the study authors evaluated all cases included, ensuring the quality of the information reported. Second, this is a preliminary report with some shortcomings (e.g., a small sample size, carried out in tertiary urban centers, where only the sensitivity of finger-prick whole blood CrAg was estimated), and formal validation studies are expected. However, we consider the results to be clinically relevant in a previously unstudied group (HIV-negative individuals with cryptococcosis). Third, we included a population predominantly with central nervous system cryptococcosis and without cases of isolated pulmonary cryptococcosis. Further studies in other subgroups of patients are necessary. The predominance of neurological manifestations reflects the current epidemiological scenario in Brazil, partially explained by the late diagnosis of cryptococcosis in HIV-negative patients. We hope that the widespread use of point-of-care tests can make it possible to change this situation. Fourth, we classified the studied population into three groups, which is a number not usually used in the literature. However, we decided to undertake this as patients with conditions or diseases classically considered to cause immunosuppression tend to be very heterogeneous. Despite the small number of patients in each category, the interpretation of the results seems not to have been affected due to the high sensitivity of the finger-prick whole blood CrAg.

In conclusion, in this study, finger-prick whole blood CrAg LFA was an important and simple diagnostic tool demonstrating high sensitivity in the diagnosis of cryptococcosis in HIV-negative patients, including those with meningitis caused by *C. gattii.* Confirmatory validation studies are necessary, including critical aspects of the spectrum disease (e.g., cases with isolated pulmonary cryptococcosis) and other developed assays (e.g., CrAg quantitative and/or semi-quantitative LFA).

## Figures and Tables

**Table 1:** Details of 30 non-HIV individuals with cryptococcosis who underwent finger-prick whole blood cryptococcal antigen lateral flow assay. Notes. M, male; F, female; LFA, lateral flow assay; LA, latex agglutination; CT, computed tomography; MRI, magnetic resonance imaging; AMB-d, deoxycholate amphotericin B; L-AMB, liposomal amphotericin-B; ABLC, amphotericin B lipid complex; *C*, *Cryptococcus*; Crypto, cryptococcal. * Histopathology examination of brain biopsy was compatible with cryptococcoma.

N	Age/Sex	Co-Morbidities	Clinical Presentation	Cryptococcus Complex Species	Finger-Prick CrAg LFA	Serum CrAg LA	Extra Neural Cryptococcal Disease	Cryptococcal Meningitis (India Ink/ CrAg/Culture)	Neuroimaging	Antifungal Induction Treatment	Outcome
**MUNOSSUPRESSED PATIENTS**											
(1)	48/M	Kidney–pancreas transplant	Headache; cough; thoracic pain	*C. neoformans*	Positive	Positive	BAL; histopathology of lung	Yes (positive/ reagent LA and positive LFA/ negative)	Leptomeningeal enhancement and dilated Virchow–Robin spaces (MRI)	ABLC plus flucytosine	Discharge to home
(2)	47/M	Stage 5 chronic kidney disease; previous kidney transplant with graft loss; hypertension; bilateral cataracts	Seizures	Not Identified	Negative	Negative	None	No (negative/not reagent LA and negative LFA/ negative) *	Single cryptococcoma (MRI)	AMB-d plus flucytosone; dexamethasone	Discharge to home
(3)	65/F	Kidney transplant; hypertension; diabetes mellitus	Headache; nausea; vomiting	Not identified	Positive	Positive	Pulmonary nodule with cavitation	Yes (positive/ reagent LA and positive LFA/ negative)	Leptomeningeal enhancement and vasculitis (MRI)	ABLC plus fluconazole	Discharge to home
(4)	48/F	Kidney transplant; hypertension	Urosepsis; Fever; mental confusion	*C. neoformans*	Positive	Negative	Pulmonary nodule; diffuse pneumonia; transplanted kidney, and bilateral adrenal glands (necropsy); blood and urine cultures	Yes (negative/not reagent LA and positive LFA/ negative)	Not performed	AMB-d plus fluconazole	In-hospital death
(5)	60/M	Cardiac transplant; diabetes mellitus	Headache	*C. neoformans*	Positive	N/A	None	Yes (positive/ reagent LA and positive LFA/ positive)	Normal imaging (MRI)	ABLC plus flucytosine	Discharge to home
(6)	51/M	Decompensated alcoholic liver cirrhosis; hepatorenal syndrome; severe thrombocytopenia	Mental confusion; oliguria; constipation	*C. neoformans*	Positive	Positive	Urine culture	Lumbar puncture contraindicated due to severe thrombocytopenia	Normal (CT)	L-AMB plus flucytosine	In-hospital death
(7)	67/M	Hepatitis C-related cirrhosis	Headache; fever; asthenia	*C. neoformans*	Positive	Negative	Blood	Yes (positive/ reagent LA and positive LFA/ positive)	Leptomeningeal enhancement (MRI)	AMB-d plus flucytosine	In-hospital death
(8)	37/M	Primary immunodeficiency; disseminated tuberculosis	Headache; mental confusion	*C. gattii*	Positive	Negative	None	Yes (negative/ reagent LA and positive LFA/ positive)	Hydrocephalus, leptomeningeal enhancement, and dilated Virchow–Robin Spaces (MRI)	AMB-d plus flucytosine	In-hospital death
(9)	45/M	Primary immunodeficiency	Headache; hemiparesis	Not identified	Positive	Negative	Bilateral pulmonary consolidations	Yes (negative/not reagent LA and positive LFA/ negative)	Cryptococcomas and perilesional edema (MRI)	ABLC plus flucytosine; dexamethasone	Discharge to home
(10)	15/F	Primary Immunodeficiency	Headache; nauseas; vomiting; mental confusion; strabismus; diplopia	*C. gattii*	Positive	Positive	Micronodular lesions in the upper lobe of right lung	Yes (positive/ reagent LA and positive LFA/ positive)	Leptomeningeal enhancement and dilated Virchow–Robin spaces (MRI)	AMB-d plus fluconazole	Discharge to home
**PATIENTS WITH OTHER COMORBIDITIES**											
(11)	86/M	Atrial tachycardia	Ulcer skin lesion in the left arm	*C. gattii*	Positive	Positive	Skin	No (negative/not reagent LA and negative LFA/ negative)	Normal (MRI)	ABLC plus flucytosine	Discharge to home
(12)	51/M	Hypertension; diabetes mellitus type 2; gout	Tumoral lesions in scalp, face, chest, and arms	*C. gattii*	Positive	Positive	Skin and pulmonary mass	LP contraindicated due to cerebellar mass	Mucinous pseudocysts and cryptococcoma (MRI)	L-AMB plus flucytosine; dexamethasone	Discharge to home
(13)	47/M	Hypertension; diabetes mellitus type 2	Headache; nausea; vomiting; hypoacusis	*C. neoformans*	Positive	N/A	None	Yes (negative/not reagent LA and positive LFA/ positive)	Leptomeningeal and ependymal enhancement, cranial nerves VII and VIII enhancement (MRI)	AMB-d plus fluconazole; prednisone	Discharge to home
(14)	31/F	Pulmonary tuberculosis	Cough weakness; fever; headache; vomiting	*C. gattii*	Positive	N/A	Pulmonary mass	Yes (negative/ reagent LA and positive LFA/ positive)	Mucinous pseudocysts and leptomeningeal enhancement (MRI)	ABLC plus flucytosine	Discharge to home
(15)	60/M	Alcoholism	Headache; mental confusion; cognitive impairment	*C. gattii*	Positive	N/A	Pulmonary mass and rib osteomyelitis	Yes (positive/ reagent LA and positive LFA/ positive)	Hydrocephalus and cryptococcoma (MRI)	L-AMB plus fluconazole	Discharge to home
(16)	47/F	Obesity	Headache; diplopia; ptosis palpebral; amaurosis; seizures	*C. gattii*	Positive	Negative	Pulmonary nodule	Yes (negative/not reagent LA and positive LFA/ positive)	Normal imaging (MRI)	AMB-d plus flucytosine	In-hospital death
(17)	57/F	Hypertension; smoker	Headache; visual abnormalities; hearing loss; hemiparesis	*C. neoformans*	Positive	Negative	Pulmonary nodule; optic papillitis	Yes (positive/ reagent LA and positive LFA/ positive)	Leptomeningeal enhancement (MRI)	ABLC plus fluconazole	Discharge to home
(18)	33/M	Tetraplegia secondary to traumatic spinal cord injury	Headache; nausea	Not identified	Positive	N/A	None	Yes (positive/ reagent LA and positive LFA/ positive)	Dilated Virchow–Robin spaces (MRI)	ABLC plus flucytosine	Discharge to home
(19)	39/M	Hypertension	Headache; fever; cognitive impairment	*C. neoformans*	Positive	Positive	None	Yes (negative/ reagent LA and positive LFA/ positive)	Communicating hydrocephalus; mucinous pseudocysts (MRI)	AMB-d plus flucytosone; prednisone	Discharge to home
(20)	39/M	Hypertension; diabetes mellitus	Headache; mental confusion	*C. gattii*	Positive	Positive	None	Yes (positive/ reagent LA and positive LFA/ positive)	Mucinous pseudocysts and leptomeningeal enhancement (MRI)	AMB-d plus flucytosine	
**APPARENTLY IMMUNOCOMPETENT PATIENTS AND THOSE WITHOUT COMORBIDITIES**											
(21)	32/F	None	Headache; papilledema; diplopia; bilateral sixth nerve palsy; unilateral hearing loss	*C. gattii*	Positive	Positive	None	Yes (positive/ reagent LA and positive LFA/ positive)	Communicating hydrocephalus; mucinous pseudocysts (MRI)	AMB-d plus fluconazole; dexamethasone; ventricular peritoneal shunt	Discharge to home
(22)	44/M	None	Fever; sweat; loss of weight; cervical adenomegaly	*C. gattii*	Positive	Positive	Neck ganglion; mediastinal mass	Yes (negative/ not reagent LA and positive LFA/ negative)	Normal (MRI)	ABLC plus flucytosine; mediastinal surgery	Discharge to home
(23)	47/M	None	Headache; retro-orbital pain; nausea; vomiting	*C. gattii*	Positive	Positive	Mediastinal lymphadenopathy	Yes (positive/ reagent LA and positive LFA/ positive)	Communicating hydrocephalus, leptomeningeal and ependymal enhancement, and dilated Virchow–Robin spaces (MRI)	ABLC plus fluconazole; dexamethasone	Discharge to home
(24)	65/M	None	Headache; diplopia; hypoacusis; mental confusion; seizures; vomiting	*C. neoformans*	Positive	N/A	Bilateral pulmonary consolidations	Yes (positive/ reagent LA and positive LFA/ positive)	Communicating hydrocephalus, leptomeningeal and ependymal enhancement (MRI)	AMB-d B plus flucytosone	Discharge to home
(26)	39/F	None	Headache; cognitive impairment; cerebellar ataxia	*C. gattii*	Positive	N/A	None	Yes (negative/not reagent LA and positive LFA/ positive)	Communicating hydrocephalus; leptomeningeal and ependymal enhancement, and mucinous pseudocysts (MRI)	AMB-d plus flucytosone; prednisone	Discharge to home
(27)	47/M	None	Headache; mental confusion	*C. gattii*	Positive	N/A	Bilateral interstitial infiltrate	Yes (positive/ reagent LA and positive LFA/ positive)	Mucinous pseudocysts (MRI)	L-AMB plus flucytosine	Discharge to home
(28)	38/F	None	Headache; mental confusion; nausea; vomiting	*C. gattii*	Positive	Positive	None	Yes (positive/ reagent LA and positive LFA/ positive)	Leptomeningeal enhancement and dilated Virchow–Robin spaces (MRI)	ABLC plus flucytosine	Discharge to home
(29)	62/F	None	Headache; nausea; vomiting; mental confusion	*C. neoformans*	Positive	Negative	None	Yes (positive/ reagent LA and positive LFA/ positive)	Dilated Virchow–Robin spaces, hypertensive hydrocephalus, leptomeningeal and ependymal enhancement (MRI)	L-AMB plus fluconazole	Discharge to home
(30)	45/F	None	Headache; mental confusion; nausea; vomiting	*C. gattii*	Positive	Negative	None	Yes (positive/ reagent LA and positive LFA/ positive)	Hypertensive hydrocephalus, leptomeningeal and ependymal enhancement (MRI)	L-AMB plus flucytosine	In-hospital death

## Data Availability

Data are contained within the article.
